# 2-Amino-6-(2,6-difluoro­benzamido)­pyridinium chloride

**DOI:** 10.1107/S1600536810029624

**Published:** 2010-07-31

**Authors:** Mohammad T. M. Al-Dajani, Nornisah Mohamed, Habibah A. Wahab, Chin Sing Yeap, Hoong-Kun Fun

**Affiliations:** a School of Pharmaceutical Sciences, Universiti Sains Malaysia, 11800 USM, Penang, Malaysia; b Malaysian Institute of Pharmaceuticals and Nutraceuticals, Ministry of Science, Technology and Innovation, Block A, 10 Persiaran Bukit Jambul, 11900 Bayan Lepas, Penang, Malaysia; c X-ray Crystallography Unit, School of Physics, Universiti Sains Malaysia, 11800 USM, Penang, Malaysia

## Abstract

In the cation of the title compound, C_12_H_10_F_2_N_3_O^+^·Cl^−^, the dihedral angle between the pyridine and benzene rings is 16.1 (1)°. In the crystal structure, mol­ecules linked into two-dimensional sheets parallel to the *bc* plane by inter­molecular N—H⋯Cl, C—H⋯Cl and C—H⋯F hydrogen bonds.

## Related literature

For general background to 2,6-diflorobenzyl­chloride derivatives, see: Beavo (1995 ▶); Beavo & Reifsnyder (1990 ▶); Hidaka & Asano (1976 ▶); Nicholson *et al.* (1991 ▶). For the stability of the temperature controller used in the data collection, see: Cosier & Glazer (1986 ▶).
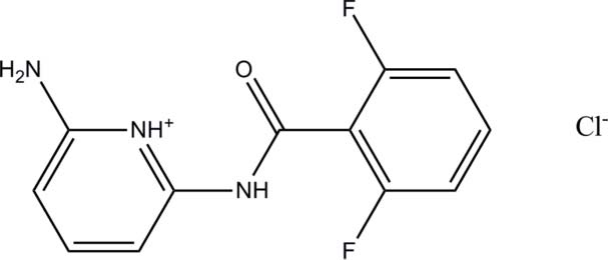


## Experimental

### 

#### Crystal data

C_12_H_10_F_2_N_3_O^+^·Cl^−^
*M* *_r_* = 285.68
Monoclinic, 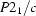

*a* = 7.3196 (2) Å
*b* = 13.6314 (3) Å
*c* = 12.2892 (3) Å
β = 99.755 (1)°
*V* = 1208.44 (5) Å^3^
*Z* = 4
Mo *K*α radiation
μ = 0.34 mm^−1^
*T* = 100 K
0.34 × 0.12 × 0.08 mm

#### Data collection

Bruker SMART APEXII CCD area-detector diffractometer
Absorption correction: multi-scan (*SADABS*; Bruker, 2009 ▶) *T*_min_ = 0.895, *T*_max_ = 0.972
11996 measured reflections
3524 independent reflections
2628 reflections with *I* > 2σ(*I*)
*R*_int_ = 0.041

#### Refinement

*R*[*F* ^2^ > 2σ(*F* ^2^)] = 0.047
*wR*(*F* ^2^) = 0.113
*S* = 1.07
3524 reflections
188 parameters
H atoms treated by a mixture of independent and constrained refinement
Δρ_max_ = 0.37 e Å^−3^
Δρ_min_ = −0.35 e Å^−3^

### 

Data collection: *APEX2* (Bruker, 2009 ▶); cell refinement: *SAINT* (Bruker, 2009 ▶); data reduction: *SAINT*; program(s) used to solve structure: *SHELXTL* (Sheldrick, 2008 ▶); program(s) used to refine structure: *SHELXTL*; molecular graphics: *SHELXTL*; software used to prepare material for publication: *SHELXTL* and *PLATON* (Spek, 2009 ▶).

## Supplementary Material

Crystal structure: contains datablocks global, I. DOI: 10.1107/S1600536810029624/lh5090sup1.cif

Structure factors: contains datablocks I. DOI: 10.1107/S1600536810029624/lh5090Isup2.hkl

Additional supplementary materials:  crystallographic information; 3D view; checkCIF report

## Figures and Tables

**Table 1:** Hydrogen-bond geometry (Å, °)

*D*—H⋯*A*	*D*—H	H⋯*A*	*D*⋯*A*	*D*—H⋯*A*
N1—H1*N*1⋯Cl1^i^	0.84 (3)	2.35 (2)	3.1622 (18)	163 (2)
N2—H1*N*2⋯Cl1	0.87 (2)	2.41 (2)	3.1678 (17)	146 (2)
N3—H1*N*3⋯Cl1^ii^	0.84 (2)	2.39 (2)	3.2140 (17)	166 (2)
N3—H2*N*3⋯Cl1	0.84 (2)	2.51 (2)	3.2346 (18)	145 (2)
C3—H3*A*⋯F2^iii^	0.93	2.52	3.414 (3)	162
C10—H10*A*⋯Cl1^iv^	0.93	2.74	3.581 (2)	151

Symmetry codes: (i) 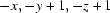; (ii) 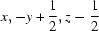; (iii) 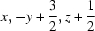; (iv) 
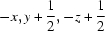
.

**Fig. 1.**
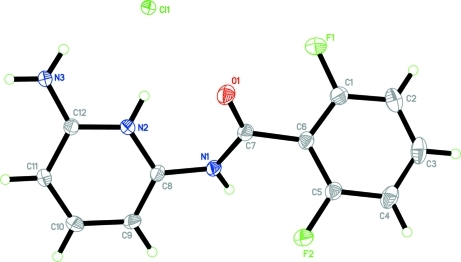
The molecular structure of the title compound with atom labels and 50% probability ellipsoids for non-H atoms.

**Fig. 2.**
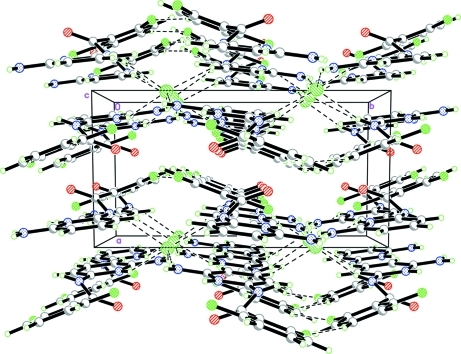
The crystal packing of title compound, viewed down the c axis, showing two 2-D planes parallel to bc plane.

## supplementary crystallographic information

### Comment

Thr derivatives of 2,6-diflorobenzylchloride involved in the inhibition of phosphodiesterases (PDEs) are enzymes which catalyze PDEs. These derivatives are classified into seven families, five of which, PDE1–PDE5, have been characterized (Beavo, 1995). The hydrolysis of cyclic nucleotides was evaluated according to the methods of Beavo & Reifsnyder (1990); Hidaka & Asano, (1976); Nicholson *et al.* (1991).

The asymmetric unit of the title compound contains one protonated 2-amino-6-(2,6-difluorobenzamido)pyridin-1-ium cation and one chloride anion (Fig. 1). The cation molecule is twisted with the dihedral angle between the pyridine ring and the benzene ring being 16.1 (1)°. In the crystal structure, molecules are linked into infinite chains along *c* axis by intermolecular C3—H3A···F2 hydrogen bonds. The chloride anions link these chains into two-dimensional sheets parallel to the *bc* plane by intermolecular N—H···Cl and C—H···Cl hydrogen bonds (Fig. 2, Table 1).

### Experimental

2,6-Difluorobenzylchloride (0.01 mol, 1.7 g) was added drop-wise into a round bottom flask containing 25 ml mixture of tetrahydrofuran (THF) and 2,6-diamino pyridine (0.01 mol, 1.1 g) with stirring. The mixture was then refluxed for two and a half hours. The oily precipitate formed was filtrated and dissolved in water and then filtrated and evaporated. The green precipitate formed was dissolved in methanol. Green needle-shaped crystals which were formed at room temperature overnight and were filtrated and dried at 333 K.

### Refinement

The N-bound hydrogen atoms were located from a difference Fourier map and refined freely. The remaining H atoms were positioned geometrically [C–H = 0.93 Å and refined using a riding model, with *U*_iso_(H) = 1.2*U*_eq_(C)].

### Crystal data

**Table:**

C_12_H_10_F_2_N_3_O^+^·Cl^−^	*F*(000) = 584
*M**_r_* = 285.68	*D*_x_ = 1.570 Mg m^−^^3^
Monoclinic, *P*2_1_/*c*	Mo *K*α radiation, λ = 0.71073 Å
Hall symbol: -P 2ybc	Cell parameters from 3017 reflections
*a* = 7.3196 (2) Å	θ = 3.4–30.0°
*b* = 13.6314 (3) Å	µ = 0.34 mm^−^^1^
*c* = 12.2892 (3) Å	*T* = 100 K
β = 99.755 (1)°	Needle, green
*V* = 1208.44 (5) Å^3^	0.34 × 0.12 × 0.08 mm
*Z* = 4	

### Data collection

**Table:**

Bruker SMART APEXII CCD area-detector diffractometer	3524 independent reflections
Radiation source: fine-focus sealed tube	2628 reflections with *I* > 2σ(*I*)
graphite	*R*_int_ = 0.041
φ and ω scans	θ_max_ = 30.0°, θ_min_ = 2.3°
Absorption correction: multi-scan (*SADABS*; Bruker, 2009)	*h* = −7→10
*T*_min_ = 0.895, *T*_max_ = 0.972	*k* = −15→19
11996 measured reflections	*l* = −17→17

### Refinement

**Table:**

Refinement on *F*^2^	Primary atom site location: structure-invariant direct methods
Least-squares matrix: full	Secondary atom site location: difference Fourier map
*R*[*F*^2^ > 2σ(*F*^2^)] = 0.047	Hydrogen site location: inferred from neighbouring sites
*wR*(*F*^2^) = 0.113	H atoms treated by a mixture of independent and constrained refinement
*S* = 1.07	*w* = 1/[σ^2^(*F*_o_^2^) + (0.0421*P*)^2^ + 0.7252*P*] where *P* = (*F*_o_^2^ + 2*F*_c_^2^)/3
3524 reflections	(Δ/σ)_max_ < 0.001
188 parameters	Δρ_max_ = 0.37 e Å^−^^3^
0 restraints	Δρ_min_ = −0.35 e Å^−^^3^

### Special details

**Table:**

Experimental. The crystal was placed in the cold stream of an Oxford Cryosystems Cobra open-flow nitrogen cryostat (Cosier & Glazer, 1986) operating at 100.0 (1) K.
Geometry. All e.s.d.'s (except the e.s.d. in the dihedral angle between two l.s. planes) are estimated using the full covariance matrix. The cell e.s.d.'s are taken into account individually in the estimation of e.s.d.'s in distances, angles and torsion angles; correlations between e.s.d.'s in cell parameters are only used when they are defined by crystal symmetry. An approximate (isotropic) treatment of cell e.s.d.'s is used for estimating e.s.d.'s involving l.s. planes.
Refinement. Refinement of *F*^2^ against ALL reflections. The weighted *R*-factor *wR* and goodness of fit *S* are based on *F*^2^, conventional *R*-factors *R* are based on *F*, with *F* set to zero for negative *F*^2^. The threshold expression of *F*^2^ > σ(*F*^2^) is used only for calculating *R*-factors(gt) *etc*. and is not relevant to the choice of reflections for refinement. *R*-factors based on *F*^2^ are statistically about twice as large as those based on *F*, and *R*- factors based on ALL data will be even larger.

### Fractional atomic coordinates and isotropic or equivalent isotropic displacement parameters (Å^2^)

**Table:**

	*x*	*y*	*z*	*U*_iso_*/*U*_eq_	
Cl1	0.00538 (7)	0.25071 (3)	0.48845 (3)	0.02010 (13)	
F1	0.25530 (18)	0.41301 (9)	0.78112 (10)	0.0280 (3)	
F2	0.45736 (17)	0.69690 (9)	0.61851 (10)	0.0250 (3)	
O1	0.3723 (2)	0.41722 (10)	0.57487 (11)	0.0235 (3)	
N1	0.2196 (2)	0.55280 (13)	0.49862 (13)	0.0179 (3)	
N2	0.1692 (2)	0.42610 (12)	0.36587 (13)	0.0174 (3)	
N3	0.1129 (3)	0.29372 (13)	0.24827 (14)	0.0212 (4)	
C1	0.3195 (3)	0.50617 (15)	0.78901 (16)	0.0209 (4)	
C2	0.3485 (3)	0.55016 (18)	0.89145 (16)	0.0267 (5)	
H2A	0.3219	0.5170	0.9530	0.032*	
C3	0.4181 (3)	0.64466 (18)	0.90068 (17)	0.0284 (5)	
H3A	0.4396	0.6750	0.9695	0.034*	
C4	0.4564 (3)	0.69494 (16)	0.80912 (17)	0.0245 (4)	
H4A	0.5063	0.7578	0.8156	0.029*	
C5	0.4181 (3)	0.64856 (15)	0.70798 (16)	0.0193 (4)	
C6	0.3492 (3)	0.55372 (14)	0.69319 (15)	0.0175 (4)	
C7	0.3170 (3)	0.50038 (14)	0.58476 (15)	0.0176 (4)	
C8	0.1933 (3)	0.52380 (14)	0.38828 (15)	0.0164 (4)	
C9	0.1878 (3)	0.58888 (15)	0.30421 (16)	0.0195 (4)	
H9A	0.2019	0.6557	0.3187	0.023*	
C10	0.1605 (3)	0.55403 (15)	0.19510 (16)	0.0208 (4)	
H10A	0.1580	0.5981	0.1371	0.025*	
C11	0.1375 (3)	0.45590 (15)	0.17297 (15)	0.0188 (4)	
H11A	0.1215	0.4333	0.1006	0.023*	
C12	0.1384 (3)	0.38928 (14)	0.26127 (14)	0.0164 (4)	
H1N1	0.181 (3)	0.6086 (19)	0.512 (2)	0.031 (7)*	
H1N2	0.166 (3)	0.3855 (18)	0.420 (2)	0.027 (6)*	
H1N3	0.095 (3)	0.2722 (18)	0.183 (2)	0.030 (7)*	
H2N3	0.105 (3)	0.2579 (18)	0.303 (2)	0.025 (6)*	

### Atomic displacement parameters (Å^2^)

**Table:**

	*U*^11^	*U*^22^	*U*^33^	*U*^12^	*U*^13^	*U*^23^
Cl1	0.0327 (3)	0.0136 (2)	0.01412 (19)	0.00100 (19)	0.00408 (17)	0.00081 (17)
F1	0.0345 (7)	0.0215 (7)	0.0282 (6)	−0.0066 (5)	0.0053 (5)	0.0033 (5)
F2	0.0321 (7)	0.0177 (6)	0.0235 (6)	−0.0045 (5)	−0.0004 (5)	0.0025 (5)
O1	0.0313 (8)	0.0155 (7)	0.0210 (7)	0.0069 (6)	−0.0038 (6)	−0.0023 (6)
N1	0.0250 (9)	0.0120 (8)	0.0155 (7)	0.0029 (7)	0.0005 (6)	−0.0011 (6)
N2	0.0245 (9)	0.0145 (8)	0.0130 (7)	−0.0002 (7)	0.0027 (6)	0.0010 (6)
N3	0.0359 (10)	0.0154 (9)	0.0124 (7)	−0.0024 (7)	0.0048 (7)	−0.0008 (7)
C1	0.0200 (10)	0.0200 (11)	0.0222 (9)	0.0002 (8)	0.0020 (8)	0.0003 (8)
C2	0.0256 (11)	0.0372 (13)	0.0173 (9)	0.0028 (10)	0.0037 (8)	−0.0016 (9)
C3	0.0273 (12)	0.0364 (13)	0.0205 (9)	0.0056 (10)	0.0009 (8)	−0.0120 (9)
C4	0.0244 (11)	0.0202 (11)	0.0271 (10)	0.0047 (8)	−0.0011 (8)	−0.0085 (8)
C5	0.0214 (10)	0.0160 (10)	0.0193 (9)	0.0018 (8)	0.0006 (7)	−0.0013 (7)
C6	0.0189 (9)	0.0160 (10)	0.0166 (8)	0.0025 (7)	−0.0002 (7)	−0.0019 (7)
C7	0.0181 (9)	0.0167 (10)	0.0170 (8)	−0.0012 (8)	0.0003 (7)	−0.0007 (7)
C8	0.0175 (9)	0.0148 (10)	0.0159 (8)	0.0007 (7)	0.0001 (7)	−0.0025 (7)
C9	0.0244 (10)	0.0127 (10)	0.0208 (9)	−0.0005 (8)	0.0019 (8)	0.0008 (7)
C10	0.0255 (10)	0.0183 (10)	0.0180 (8)	−0.0001 (8)	0.0025 (8)	0.0046 (8)
C11	0.0233 (10)	0.0199 (10)	0.0130 (8)	−0.0012 (8)	0.0024 (7)	−0.0005 (7)
C12	0.0186 (9)	0.0152 (9)	0.0150 (8)	0.0007 (7)	0.0017 (7)	−0.0007 (7)

### Geometric parameters (Å, °)

**Table:**

F1—C1	1.352 (2)	C2—H2A	0.9300
F2—C5	1.354 (2)	C3—C4	1.386 (3)
O1—C7	1.217 (2)	C3—H3A	0.9300
N1—C7	1.373 (2)	C4—C5	1.380 (3)
N1—C8	1.394 (2)	C4—H4A	0.9300
N1—H1N1	0.84 (3)	C5—C6	1.388 (3)
N2—C12	1.363 (2)	C6—C7	1.501 (3)
N2—C8	1.365 (2)	C8—C9	1.357 (3)
N2—H1N2	0.87 (2)	C9—C10	1.405 (3)
N3—C12	1.322 (3)	C9—H9A	0.9300
N3—H1N3	0.84 (3)	C10—C11	1.370 (3)
N3—H2N3	0.84 (2)	C10—H10A	0.9300
C1—C2	1.378 (3)	C11—C12	1.414 (3)
C1—C6	1.393 (3)	C11—H11A	0.9300
C2—C3	1.383 (3)		
			
C7—N1—C8	124.78 (17)	C4—C5—C6	123.94 (19)
C7—N1—H1N1	117.9 (17)	C5—C6—C1	115.33 (17)
C8—N1—H1N1	117.0 (17)	C5—C6—C7	124.48 (17)
C12—N2—C8	123.00 (16)	C1—C6—C7	120.10 (18)
C12—N2—H1N2	117.7 (16)	O1—C7—N1	123.10 (18)
C8—N2—H1N2	119.2 (16)	O1—C7—C6	122.36 (17)
C12—N3—H1N3	117.0 (17)	N1—C7—C6	114.54 (17)
C12—N3—H2N3	120.1 (16)	C9—C8—N2	119.86 (17)
H1N3—N3—H2N3	123 (2)	C9—C8—N1	122.45 (18)
F1—C1—C2	118.17 (18)	N2—C8—N1	117.68 (16)
F1—C1—C6	118.59 (17)	C8—C9—C10	119.13 (18)
C2—C1—C6	123.2 (2)	C8—C9—H9A	120.4
C1—C2—C3	118.5 (2)	C10—C9—H9A	120.4
C1—C2—H2A	120.7	C11—C10—C9	120.84 (18)
C3—C2—H2A	120.7	C11—C10—H10A	119.6
C2—C3—C4	121.09 (19)	C9—C10—H10A	119.6
C2—C3—H3A	119.5	C10—C11—C12	119.36 (17)
C4—C3—H3A	119.5	C10—C11—H11A	120.3
C5—C4—C3	117.8 (2)	C12—C11—H11A	120.3
C5—C4—H4A	121.1	N3—C12—N2	118.32 (17)
C3—C4—H4A	121.1	N3—C12—C11	123.91 (17)
F2—C5—C4	118.02 (18)	N2—C12—C11	117.77 (17)
F2—C5—C6	117.99 (16)		
			
F1—C1—C2—C3	178.58 (19)	C5—C6—C7—O1	131.1 (2)
C6—C1—C2—C3	−2.9 (3)	C1—C6—C7—O1	−45.2 (3)
C1—C2—C3—C4	0.7 (3)	C5—C6—C7—N1	−49.3 (3)
C2—C3—C4—C5	1.7 (3)	C1—C6—C7—N1	134.4 (2)
C3—C4—C5—F2	−179.22 (18)	C12—N2—C8—C9	−0.2 (3)
C3—C4—C5—C6	−2.1 (3)	C12—N2—C8—N1	178.56 (18)
F2—C5—C6—C1	177.18 (17)	C7—N1—C8—C9	−144.7 (2)
C4—C5—C6—C1	0.1 (3)	C7—N1—C8—N2	36.5 (3)
F2—C5—C6—C7	0.7 (3)	N2—C8—C9—C10	−1.2 (3)
C4—C5—C6—C7	−176.40 (19)	N1—C8—C9—C10	−179.89 (18)
F1—C1—C6—C5	−178.98 (17)	C8—C9—C10—C11	0.8 (3)
C2—C1—C6—C5	2.5 (3)	C9—C10—C11—C12	1.0 (3)
F1—C1—C6—C7	−2.4 (3)	C8—N2—C12—N3	−178.62 (18)
C2—C1—C6—C7	179.1 (2)	C8—N2—C12—C11	2.0 (3)
C8—N1—C7—O1	−9.0 (3)	C10—C11—C12—N3	178.30 (19)
C8—N1—C7—C6	171.45 (18)	C10—C11—C12—N2	−2.3 (3)

### Hydrogen-bond geometry (Å, °)

**Table:**

*D*—H···*A*	*D*—H	H···*A*	*D*···*A*	*D*—H···*A*
N1—H1N1···Cl1^i^	0.84 (3)	2.35 (2)	3.1622 (18)	163 (2)
N2—H1N2···Cl1	0.87 (2)	2.41 (2)	3.1678 (17)	146 (2)
N3—H1N3···Cl1^ii^	0.84 (2)	2.39 (2)	3.2140 (17)	166 (2)
N3—H2N3···Cl1	0.84 (2)	2.51 (2)	3.2346 (18)	145 (2)
C3—H3A···F2^iii^	0.93	2.52	3.414 (3)	162
C10—H10A···Cl1^iv^	0.93	2.74	3.581 (2)	151

Symmetry codes: (i) −*x*, −*y*+1, −*z*+1; (ii) *x*, −*y*+1/2, *z*−1/2; (iii) *x*, −*y*+3/2, *z*+1/2; (iv) −*x*, *y*+1/2, −*z*+1/2.
